# Nutraceutical Potential of *Sideroxylon cinereum*, an Endemic Mauritian Fruit of the Sapotaceae Family, Through the Elucidation of Its Phytochemical Composition and Antioxidant Activity

**DOI:** 10.3390/molecules30143041

**Published:** 2025-07-20

**Authors:** Cheetra Bhajan, Joyce Govinden Soulange, Vijayanti Mala Ranghoo-Sanmukhiya, Remigiusz Olędzki, Daniel Ociński, Irena Jacukowicz-Sobala, Adam Zając, Melanie-Jayne R. Howes, Joanna Harasym

**Affiliations:** 1 Department of Agricultural and Food Science, University of Mauritius, Reduit 80835, Mauritius; cheetrabhajan@gmail.com (C.B.); joyces@uom.ac.mu (J.G.S.); m.sanmukhiya@uom.ac.mu (V.M.R.-S.); 2 Department of Biotechnology and Food Analysis, Wroclaw University of Economics and Business, Komandorska 118/120, 53-345 Wroclaw, Poland; remigiusz.oledzki@ue.wroc.pl; 3 Adaptive Food Systems Accelerator-Science Centre, Wroclaw University of Economics and Business, Komandorska 118/120, 53-345 Wroclaw, Poland; 4 Department of Chemical Technology, Wroclaw University of Economics and Business, Komandorska 118/120, 53-345 Wrocław, Poland; daniel.ocinski@ue.wroc.pl (D.O.); irena.jacukowicz-sobala@ue.wroc.pl (I.J.-S.); 5 Department of Bioorganic Chemistry, Wroclaw University of Economics and Business, Komandorska 118/120, 53-345 Wrocław, Poland; adam.zajac@ue.wroc.pl; 6 Royal Botanic Gardens Kew, Surrey TW9 3AB, UK; m.howes@kew.org

**Keywords:** Sapotaceae, Mauritius, *Sideroxylon cinereum*, endemic fruit, phytochemicals, antioxidant activity, nutraceutical

## Abstract

*Sideroxylon cinereum*, an endemic Mauritian fruit, was investigated through comprehensive chemical analyses of solvent extracts from its pulp and seed. Dried fruit materials were subjected to maceration using water and organic solvents including methanol, ethanol, propanol, and acetone to obtain extracts of varying polarity. Preliminary phytochemical screening revealed the presence of several bioactive compounds, with pulp extracts generally richer in phytochemicals than seed extracts. UV-Vis and FTIR analyses confirmed key organic constituents, including sulfoxides in seeds. HPLC quantification showed notable citric acid content in the pulp (15.63 mg/g dry weight). Antioxidant assays indicated that organic solvent extracts of the pulp had superior free radical scavenging activity, while the seed’s aqueous extract exhibited the highest ferric reducing power. GC–MS profiling identified a diverse bioactive profile rich in terpenes, notably lanosterol acetate (>45% in both pulp and seeds). It is important to note that these findings are based on solvent extracts, which may differ from the phytochemical composition of the whole fruit as typically consumed. Among the extracts, aqueous fractions are likely the most relevant to dietary intake. Overall, the extracts of *Sideroxylon cinereum* pulp and seed show potential as sources of bioactive compounds for functional product development.

## 1. Introduction

*Sideroxylon cinereum* Lam., commonly known as ‘manglier vert,’ is a Mauritian endemic plant belonging to the Sapotaceae family, a part of the Ericales order. The Sapotaceae family consists of 1250 species of trees and shrubs, categorised into 53 genera and 5 tribes. The presence of latex, simple and entire leaves, malpighiaceous trichomes, flowers arranged in fascicles, and edible fruits are all important diagnostic features of the family [1]. Many fruit-producing members of Sapotaceae plants have been reported as valuable sources of nutritional and medicinal active compounds with antioxidant, anti-inflammatory, antiulcer, antimicrobial, anti-hypercholesterolemic, and immunomodulatory activities among others [2].

Various Sapotaceae species are economically significant resources of high-quality timber and popular tropical fruits such as the sapodilla (*Manilkara zapota*) and star apple (*Chrysophyllum cainito*). Two particularly valuable African species of the family are *Vitellaria paradoxa*, used for shea butter production, and *Argania spinosa*, used for argan oil production [3]. The family is well known for its diverse bioactive compounds, primarily terpenes. The rich phytochemical profile, found in the edible parts of the plants, makes the Sapotaceae family a resource with enormous potential for food applications. Plants in the family naturally contain a high concentration of antioxidants in their various parts, particularly the fruits. This characteristic can be attributed to the high level of terpenes such as the tetraterpenoid carotenoids [1]. Therefore, researchers’ interest in various Sapotaceae species has been piqued due to the terpenoid content.

Phytochemical studies have been conducted on several genera within the Sapotaceae family, including *Mimusops*, *Manilkara*, *Argania*, *Sideroxylon*, and *Vitellaria*, where approximately 100 triterpenoid compounds have been identified [4]. The main triterpenoids found in Sapotaceae plants include protobassic acid, 16-hydroxyprotobassic acid, bayogenin, and oleanolic acid derivatives, typically conjugated with monodesmosidic and bidesmosidic sugar side chains [1]. These triterpenoid aglycones are commonly glycosylated with various sugar moieties such as xylose, rhamnose, arabinose, apiose, and glucuronic acid—a sugar acid derivative [5]. It is important to note that organic acids like citric and malic acid are a distinct class of compounds and not directly attached to the triterpenoid structures. These bioactive compounds, including glycosylated triterpenoids and organic acids, may contribute nutritional and functional benefits, making them valuable resources for food product development [6].

Sideroxylon capiri (Tempisque), a plant in the genus *Sideroxylon* of the Sapotaceae family, produces palatable fruits that are used as a food condiment and to cure some kidney disorders. Percolation-derived methanol extracts of *S. capiri* contain phenols, flavonoids, steroids, and tannins. Furthermore, the extract was determined to be harmless [7]. A more recent study found phytochemicals such as quercetin, kaempferol, and gallic acid in the plant at concentrations of 230–240 µg/g dry weight when subjected to stressful conditions such as UV–B Radiation [8].

The natural habitat of *S. cinereum* is Mauritius, a tropical island southeast of Africa. Mauritius is a part of the Mascarene Islands in the Indian Ocean, about 800 km east of Madagascar. Approximately 600 plant species have been documented, with 277 being endemic to the island and 147 exclusively found in the Mascarene Islands.

Among them, *S. cinereum* is a medium-sized, evergreen tree, typically reaching heights of 10–15 m. This tree occurs primarily in remnant native forests, preferring well-drained soils and the humid conditions of Mauritius. It has a slender trunk that may occasionally divide near the base, and rough, greyish-brown bark. The leaves are alternate, simple, elliptic to oblong, coriaceous, and globous, with entire margins, a prominent midrib, and finely reticulate venation. The inflorescence, which consists of densely packed small cream-coloured flowers, grows directly on the branches. These hermaphroditic flowers bear fruit which are spherical in shape and have a globose seed. The fruits of the species *S. cinereum* have been consumed locally for decades for their pleasant flavour and sweet taste with no adverse health effects.

While ethnopharmacological records are limited, related species within the *Sideroxylon* genus have been traditionally used to treat inflammation, infections, and wounds. Recent studies have begun to explore the pharmacological potential of *S. cinereum* itself. Notably in a study conducted by Kauroo et al. [9], on the pluripharmacological potential of Mascarene endemic plants, the anti-cancer and immunomodulatory activities of the leaves of the plant were investigated. *S. cinereum* leaf extracts demonstrated moderate to low toxicity against non-tumorigenic kidney and keratinocyte cell lines. LC–MS profiling of the extracts revealed the presence of flavonoids, phenolic acids, triterpenoids, and fatty acids—classes of compounds frequently associated with antioxidant and anti-inflammatory effects.

The antioxidant potential of *S. cinereum* likely arises from a multifaceted synergy of its phenolic and triterpenoid contents. These compounds act through both direct free radical quenching and indirect modulation of antioxidant pathways, suggesting therapeutic promise for managing oxidative stress-related disorders. Detailed phytochemical and mechanistic studies are warranted to confirm the presence and roles of these bioactive compounds in *S. cinereum* [10]. Therefore, the presence of these compounds in *S. cinereum* leaves suggests antioxidant potential; however, no studies to date have evaluated the antioxidant properties or mechanisms of action in the fruit and seed. Our work addresses this gap by characterizing these parts and examining their antioxidant activity in detail which may support its nutritional and pharmacological valorisation.

Exploratory studies have been conducted recently to identify endemic wild fruits as promising crops with high economic value. Gathering scientific evidence of the properties of these wild resources is critical for assessing their contribution to local biodiversity and their effects on nutrition and human health [11]. As a result, expanding this species’ chemical and biological knowledge would add value to its nutraceutical and commercialization potential. Fruit seeds may have high concentrations of bioactive substances with potential applications in the culinary industries and may help to meet the current demand for natural ingredients, which are generally preferred because they have fewer side effects than artificial compounds [12]. In this context, the current study aimed to identify the phytochemical profiles and contents of *S. cinereum* fruit pulp and seeds and assess their antioxidant properties. This information will highlight their potential use in developing functional foods and preserving and promoting their use as traditional foods.

## 2. Results

### 2.1. Preliminary Qualitative Phytochemical Screening

The results from the qualitative phytochemical analysis of the extracts are shown in Table 1. Phenols were present in all samples except the seed water extract, with the methanolic and ethanolic pulp extracts containing many of these compounds (up to 102.5 GAE mg/g DM). Flavonoids were not detected in the pulp extracts but were moderately detected in the seed extracts (up to 9.45 QE mg/g DM). Terpenes were abundant in the pulp but were not detected in seeds (up to 697.5 LE mg/g DM). Coumarins and tannins were present in moderate amounts in all organic solvent extracts. All extracts contained alkaloids, excluding the seed water extract. Reducing sugars were detected only in water extracts, while proteins were found in water, ethanol, and acetone extracts.

### 2.2. Quantitative Phytochemical Analysis

**Figure 1:**
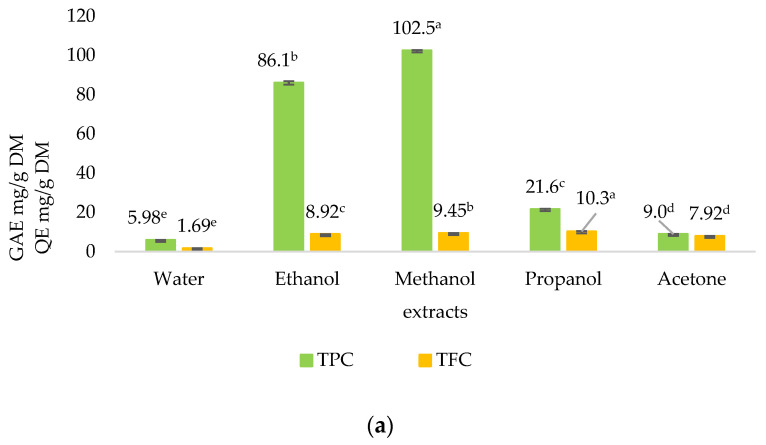

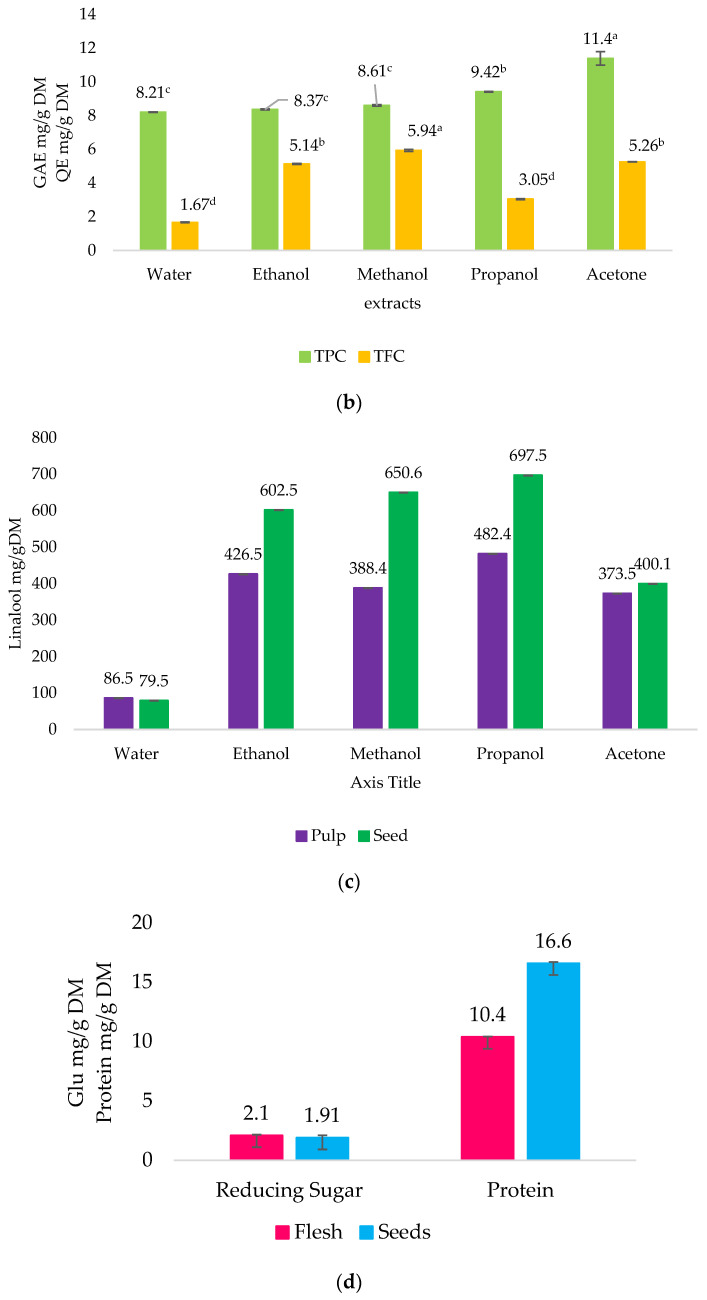
Total polyphenol content (TPC) and total flavonoid content (TFC) of (**a**) pulp extracts and (**b**) seed extracts. (**c**) Total terpenoid content (TTC). (**d**) Reducing sugar and protein content of the water extract of *S. cinereum* pulp and seeds. GAE—gallic acid equivalent, QE—quercetin equivalent, LE—linalool equivalent, Glu—glucose equivalent, DM—dry matter. Lowercase letters indicate statistically significant difference between solvent fractions (*p* = 0.05). Error bars represent the standard deviation of the mean.

Figure 1a,b show the TPC and TFC of *S. cinereum* pulp and seed extracts, respectively. The pulp extracts of *S. cinereum* had the highest (*p* < 0.05) TPC value observed in the methanol fraction, followed by the ethanol fraction.

The TPC values of most seed extracts ranged from 8 to 12 mg GAE/g DM. The TFC of the fruit pulp ranged between 0 and 11 mg QE/g DM and seed between 1 and 6 mg QE/g DM, which is almost twice as low.

The TTC of the extracts was strikingly higher, as shown in Figure 1c, when compared with TPC and TFC. The propanol fraction of the pulp produced the highest (*p* < 0.05) TTC value of 697.5 ± 0.40 mg LE/g DM.

Figure 1d shows the reducing sugars (RS) content and protein (P) content of the water extracts. The amount of RS was rather low, but protein content was fairly high compared with the RS and protein content of fruits in general. No significant difference between the pulp and seed RS can be observed.

### 2.3. UV–Vis Absorbance Characteristics

**Figure 2:**
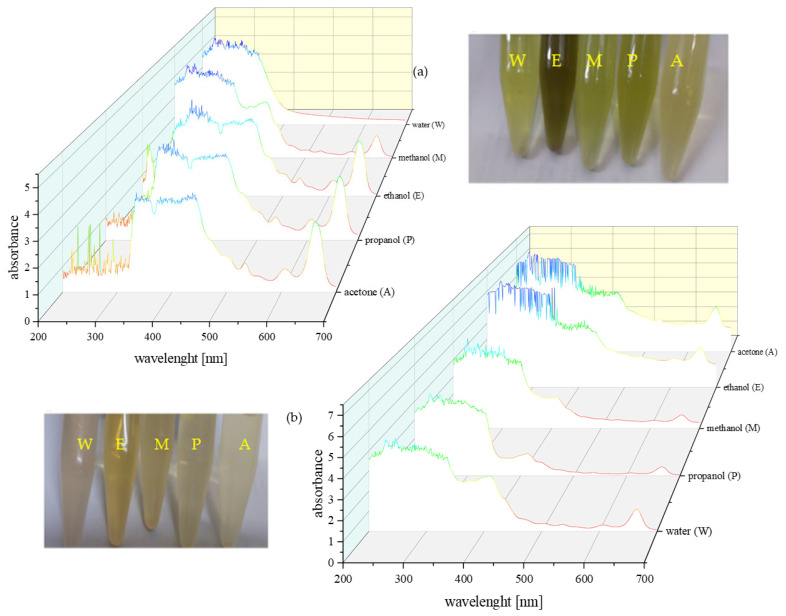
UV–Vis spectrum of different polarity solvent extracts of (**a**) *S. cinereum* pulp and (**b**) *S. cinereum* seeds.

As a result of extracting the bright green *S. cinereum* fruits, the obtained pulp extracts appeared in colours ranging from yellow to greenish-yellow and brown, while the seed extracts colours ranged from pale beige to yellow and brown (Figure 2).

The extracts from the flesh exhibited more intense UV–Vis absorption than those from the seeds. The UV–Vis spectra, recorded between 200 and 700 nm for the five different solvent extracts, displayed multiple absorption peaks. The greater colour intensity and spectral complexity of the organic solvent extracts compared with the aqueous extracts suggests a higher diversity of phytochemicals.

All samples showed absorption near 260 nm, which may be consistent with the presence of alkaloids; however, as many organic compounds absorb in this region, this observation is not definitive. Additional peaks observed in the 300–350 nm and 400–450 nm ranges could be attributed to phenolic compounds, including flavonoids and tannins, respectively. Minor peaks between 450–500 nm may indicate carotenoids, and features in the 450–550 nm region could reflect the presence of terpenoids, although these assignments remain tentative. Notably, all spectra—except for the water extract of the flesh—exhibited strong bands in the 600–700 nm range, which are commonly associated with chlorophyll [13].

These spectral features are suggestive but not conclusive; therefore, further analysis using complementary techniques (e.g., GC–MS) would be necessary to confirm the identity of the compounds responsible for these absorptions.

### 2.4. FTIR Characterization

**Figure 3:**
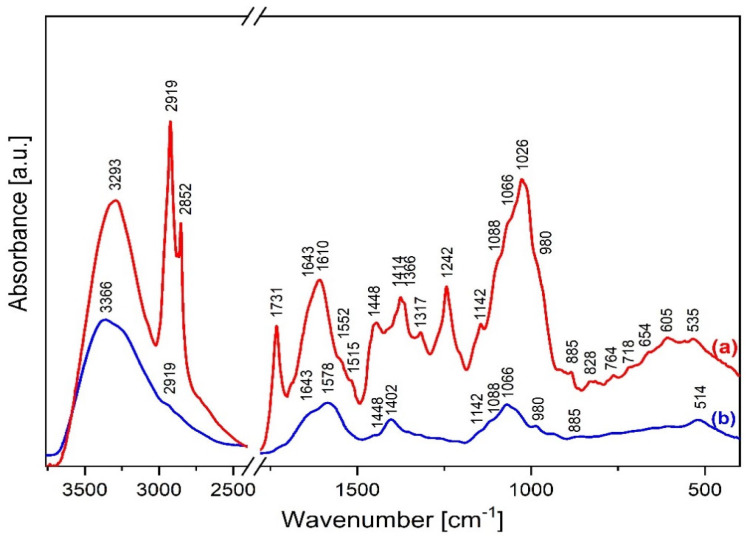
FT−MIR spectra of *S. cinereum* (a) pulp and (b) seeds.

The studies on the composition of *S. cinereum* were focused on analysing lyophilised fruit pulp and seeds, except for IR analysis. The infrared spectra in Figure 3 display peaks corresponding to the functional groups present in whole parts of the *S. cinereum* plant.

The broad absorption band at ca. 3300 cm^−1^ in the fruit spectrum corresponds to the free hydroxyl groups’ stretching υ(OH) mode and those involved in the intra- and intermolecular hydrogen bonds. The double bands of 2918 cm^−1^ and 2855 cm^−1^ indicate the vibrations of νas(CH)/CH_3_ and νs(CH)/CH_2_, respectively [14]. Spectroscopic analysis suggests a high lipid content in the fruit, including sterol, triterpene and sulpholipid derivatives. This is evidenced by sharp bands of medium intensity at the following wavenumbers: 1731 (ν(C=O)), 1643 (ν(C=C)/cis), 1448 (δ(CH)/CH_2_), 1414 (ρ(=CH)/cis), 1366 (δ(CH)/CH_3_), 1242 (ν(C-O-S), δ(-CH_2_-)), 1142 (ν(C-O), δ(-CH_2_-)), 1066 (ν(S=O), 828 ((ν(C-O-S) and 718 (ρ(-(CH_2_)n-), ρ(C=C)/-, cis) cm^−1^ [15,16,17,18]. The amide I and II bond’s characteristic vibrations appear at 1610, 1552 and 1515 cm^−1^, respectively [14]. The contour of the infrared spectrum at 1088 and 1026 cm^−1^ indicates the presence of monosaccharides in the pulp. The fingerprint region reveals the presence of a halo compound’s C–I stretching at 535 cm^−1^ [14].

The IR spectrum of the seeds indicates a poorer chemical composition than the fruit pulp. Similarly, the broad band at ca. 3300 cm^−1^ shows the presence of the intra- and inter-molecular hydrogen bonds. The infrared spectrum shows similar positions of the amide I and II bands. A slightly sharper band at 1066 cm^−1^ may indicate S=O stretching vibrations of a sulfoxide group [14].

### 2.5. Saccharides and Organic Acids Characteristics by HPLC

Sugars and organic acids comprise a group of compounds that are soluble in aqueous solutions. Consequently, they are also easily absorbed in the human gastrointestinal tract. Their composition, content in plant parts and concentration in the aqueous extracts reveal the calorific and nutritional potential of *S. cinereum*. The findings can also help evaluate its usability for processing and preparing food additives.

**Figure 4:**
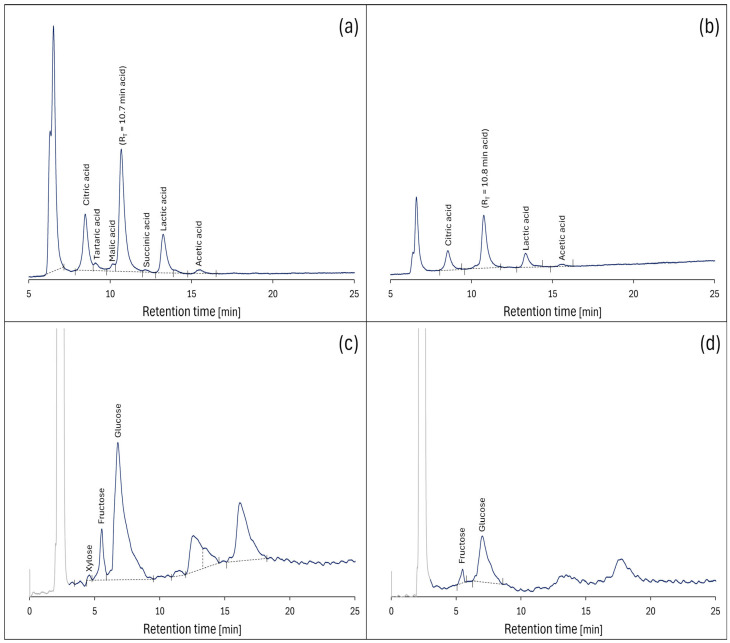
HPLC organic acids chromatogram of the *S. cinereum* water extract of (**a**) pulp, (**b**) seeds and sugars, (**c**) pulp, and (**d**) seeds.

The HPLC chromatograms of the extracts and calculated concentrations of detected organic acids and monosaccharides in the pulp and seed are presented in Figure 4 and Table 2, respectively.

As shown in Figure 4, the individual acids have been effectively separated, enabling their exact identification and quantification with statistical validation. The chromatographic analysis revealed distinct compositional hierarchies within each tissue type. In the pulp extract, glucose emerged as the most abundant compound (3.91 ± 0.20 mg/mL), appearing as a well-developed peak at 6.79 min retention time and showing significantly higher concentrations than all other detected compounds (*p* < 0.05). This was followed by citric acid (1.56 ± 0.08 mg/mL, 8.47 min) and lactic acid (1.41 ± 0.07 mg/mL, 13.26 min), which formed a second tier of abundant compounds that were significantly more concentrated than the remaining organic acids and sugars.

The seed extract demonstrated a comparable but simplified pattern, with glucose again representing the predominant compound (1.16 ± 0.01 mg/mL, 7.02 min), significantly exceeding the concentrations of citric acid (0.73 ± 0.04 mg/mL, 8.54 min) and lactic acid (0.57 ± 0.03 mg/mL, 13.34 min) (*p* < 0.05). Small quantities of tartaric acid (0.26 ± 0.01 mg/mL, 9.08 min), malic acid (0.18 ± 0.01 mg/mL, 10.15 min), and succinic acid (0.23 ± 0.01 mg/mL, 12.16 min) were detected exclusively in pulp extracts, contributing to its more complex compositional profile compared with seeds.

The well-developed peak at 10.7–10.8 min most likely resulted from monosaccharides (glucose, fructose, and xylose) in the extracts, which was confirmed by analysing saccharide standards under identical conditions where their retention times fell within a very narrow range, making complete separation challenging. The separation of monosaccharides was less effective (Figure 4c,d), but allowed for quantification of the main simple sugars present. In the pulp extract, fructose (0.54 ± 0.03 mg/mL, 5.56 min) and a trace amount of xylose (0.03 ± 0.00 mg/mL, 4.59 min) were also detected, with fructose showing significantly higher concentrations than xylose but remaining substantially lower than glucose levels.

The peaks appearing at approximately 6.5 min in both chromatograms likely result from a mixture of polyphenolic compounds characterized by complex structures of large molecules that are not effectively retained by the cation-exchange HPLC column. The last two peaks observed on the chromatograms probably resulted from cellobiose and additional polyphenolic compounds retained longer due to specific separation mechanisms of adsorbents containing amine functional groups.

Between-tissue comparisons revealed that all compounds present in both pulp and seeds showed significantly higher concentrations in pulp (*p* < 0.001). Glucose exhibited the largest absolute difference (27.49 mg/g higher in pulp), while fructose demonstrated the most pronounced relative difference (4.86-fold higher in pulp). These findings indicate that the fruit pulp serves as the primary repository for water-soluble organic compounds, consistent with its biological role in energy storage and seed dispersal attraction.

### 2.6. Compound Identification by GC–MS

Mass spectrometry is useful in plant phytochemistry because it can analyse the structure of the many phytoconstituents found in different extracts. Researchers discovered that this approach can separate chemical prints with high accuracy and precision [18].

Terpenoids can be polar, soluble, moderately volatile, and coloured based on their functional groups. Highly oxygenated, polar triterpenes, as well as triterpenoids, are typically extracted with ethanol, methanol, and water. GC–MS is thought to be appropriate for terpene analysis because it provides measurements with high consistency, dynamic range, and a universal mass spectral library for substances with low molecular weights [19,20,21]. As a result, the current work attempted to use GC–MS to analyse the phytochemicals in methanolic extracts of selected Mauritian plants.

Methanol was chosen due to its high polarity and strong solubilizing capacity for a broad spectrum of phytochemicals, including alkaloids, phenolic acids, flavonoids, steroids, and glycosides, as demonstrated in prior studies that combined methanol extraction with GC–MS profiling of plant samples for chemical characterization [22,23,24,25]

A 1 g:10 mL (*w*/*v*) ratio of plant material to methanol was chosen for the extraction process as this falls well within the commonly used range of 1:5 to 1:20 cited in phytochemical analyses. Using a 1:10 ratio provides an effective balance: it ensures sufficient solvent to extract a broad range of phytochemicals while avoiding excessive dilution that could impair GC–MS detection. Additionally, this ratio aligns well with standard laboratory workflows (e.g., using 10 mL centrifuge tubes), facilitating reproducible and scalable sample preparation [22].

**Figure 5:**
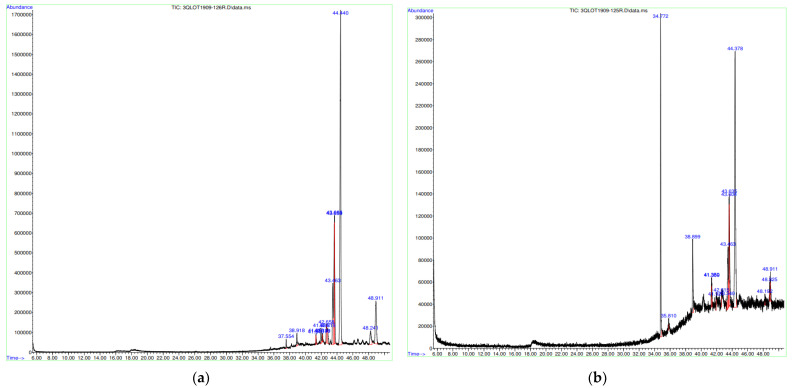
GC–MS chromatograms of (**a**) *S. cinereum* flesh and (**b**) *S. cinereum* seeds.

The GC–MS chromatogram of methanolic extracts show the presence of 14 identified compounds presented in Figure 5 and Table 3 with retention times and area percentages. The dominant constituent in both *S. cinereum* pulp and seed was lanosterol acetate. The pulp was also rich in other terpenoids, α-amyrin, perkeol acetate, chromane and friedelane. In the extract of seeds, with the exception of lanosterol acetate, other lanostan skeletons containing terpenoid were identified in large amounts, such as lanosta-7,9 (11)-dien-3-ol, acetate, (3.beta.)- and also 1,2 1,2-benzenedicarboxylic acid.

### 2.7. Antioxidant and Oxidoreductive Activity

The antioxidant and oxidoreductive characteristics of the extracts are presented in Table 4. The propanol pulp extract had the highest (*p* < 0.05) DPPH and ABTS value, while the acetone pulp extract had the highest (*p* < 0.05) FRAP value. The seed water extract had the highest FRAP value of all extracts.

### 2.8. Correlation Analysis Between Phytochemical Content and Antioxidant Activity

Table 5 shows significantly low and even negative FRAP correlations were observed between the studied phytochemical contents and antioxidant results, indicating that these compounds were not the main contributors to the antioxidant activities of the extracts. In fact, the TTC R values were the highest, especially with ABTS. The correlation analysis revealed moderate positive relationships between total phenolic content (TPC) and total flavonoid content (TFC) (r = 0.614) and between TPC and total terpenoid content (TTC) (r = 0.598). TFC and TTC showed a strong positive correlation (r = 0.875). Antioxidant activities (DPPH and ABTS) were weakly correlated with phytochemical contents: DPPH had low positive correlations with TPC (r = 0.246), TFC (r = 0.152), and TTC (r = 0.336), while ABTS showed negligible correlations with TPC (r = 0.082) and TFC (r = 0.300) but a moderate correlation with TTC (r = 0.481). Notably, FRAP exhibited weak negative correlations with TPC (r = −0.362), TFC (r = −0.395), and TTC (r = −0.365), suggesting these compounds were not the primary contributors to reducing power. The DPPH and ABTS activities were weakly inversely related (r = −0.160), while FRAP and ABTS showed no meaningful correlation (r = −0.011).

## 3. Discussion

The qualitative analysis of the extract compositions revealed that the fruit pulp contained a higher concentration of phytochemicals compared with the seeds. This observation agrees with other studies (for instance, on *Pleiogynium Timorensae*), as seeds are better sources of energy storage compounds, such as triglycerides and polysaccharides, than other phytochemicals [26]. Phytoconstituents can be extracted from various plant parts, including leaves, flowers, and fruits, each of which represents a distinct matrix with unique chemical characteristics that may influence extraction efficiency [27]. The naturally hard structures of the complex cell wall in the seeds significantly hinder the extraction of certain bioactive compounds. Their frequent linkages and networks with proteins and polysaccharides restrict this process, which may explain the richer phytochemical composition of the fruit pulp extracts [28].

Ethanol and methanol extracts yielded higher (*p* < 0.05) TPC values due to their higher polarity and better solubility for such compounds [29]. The extraction of phenolic compounds from plant materials depends on factors such as the sample matrix as well as the chemical properties of the phenolics, including their concentration, polarity, molecular structure, number of aromatic rings, and hydroxyl groups [30]. In a study on the phenolic content in *Sideroxylon capiri* fruits, the highest TPC value observed was 12.2 mg GAE/g DM [7]. The values obtained in this study are much higher, making *S. cinereum* a Mauritian fruit with remarkable amounts of phenolics.

The total phenolic content (TPC) provides a general estimate of the phenolic compounds present in the extracts but does not offer information on the specific types or structures of these compounds. Flavonoids, which are a major subclass of phenolics with well-documented antioxidant properties, were therefore quantified separately as total flavonoid content (TFC). The TFC values observed in this study were relatively low. One possible factor influencing flavonoid detection could be their interaction with plant cellular structures; for example, some flavonoids, such as quercetin, have been shown to associate with lipid bilayers due to their chemical properties [31,32]. However, this remains a hypothesis and requires further investigation with targeted extraction and localization studies.

Interestingly, when compared with values reported for six Mauritian endemic species, which ranged from 0.2 to 0.7 mg QE/g dry weight (dw) [33], the TFC values observed for *S. cinereum* in the present study were comparatively high. These variations may be influenced by species-specific phytochemistry or environmental factors such as altitude, soil composition, and climatic conditions [34].

Literature data showed that plants of the genus *S. cinereum* contain compounds such as quercetin O-glycoside, triterpenoids, and pentacyclic triterpenoids [7]. These compounds probably contribute to the high levels of polyphenols and terpenoids observed in the current study. In turn, phenolics, proteins, and sugars, can combine to form high molecular weight melanoidins. These multicomponent polymers may add antioxidative potential to the plant [35].

The identified components are consistent with previous research on the phytochemical content of the fruit. Ling & Hadinoto. [36] have elucidated that organic solvents have a level of solubility that is useful for optimizing the extraction of both hydrophilic and hydrophobic compounds. In turn, Mustafa & Chin. [37] explain that water, as a highly polar solvent with high surface tension, can wash away hydrophilic compounds with –COOH or –OH groups from plant tissues. In addition to small bioactive molecules, water extracts may also contain polysaccharides and proteins. This is reflected in the current study, as only water extracts contained saccharides, while proteins were also detected in ethanol and acetone. The analysis of aqueous extracts showed a higher protein extraction yield than reducing sugars 10.4–16.6 mg/g and 1.9–2.1 mg/g, respectively, particularly during seed treatment.

Phytochemical analyses by UV–Vis and FTIR spectroscopy point towards the content of important health-promoting chemicals in *S. cinereum*.

The FTIR spectra of fruit pulp and seeds revealed peaks characteristic of amines ubiquitous in higher plants. Mainly, amines are precursors for several alkaloids, primarily catabolized by diamine and polyamine oxidases via oxidative deamination [38]. These findings support the results of the phytochemical screening of the extracts, as they were found to be rich in alkaloids. The seeds of *S. cinereum* contained morphinan alkaloids, specifically identified through GC–MS analysis. Plants produce these physiologically active substances, which can be used as ingredients to create pharmaceutical products like analgesics [39].

An interesting result from the FTIR study was the presence of a peak indicating a sulphoxide group, which was unique in the seed spectrum. Sulphoxides are important sulphur antioxidants commonly found in Allium vegetables such as garlic, one of the oldest medicinal plants. These compounds have received much attention since their degradation products are potent anticarcinogenic compounds [40].

Furthermore, FTIR results indicated the presence of aldehyde groups. Volatile aldehydes of plants are responsible for their distinct aromas and flavours. Aldehydes usually fall into the monoterpenes category, classified into unsaturated hydrocarbons, alcohols, alcohol esters, aldehydes, and ketones [41]. Thus, the obtained results justify the high amount of terpene detected through quantitative analysis.

The HPLC analysis of the selected extracts revealed the content of monosaccharides: glucose, fructose, and xylose in the range of 0.35–39.09 mg/g DM, which is a typical value for fruits [42].

Glucose and fructose are among the sugars metabolised during fruit growth and development, and glucose is also an important constituent of polysaccharides forming cell walls, including cellulose [43]. Glucose is also abundant in fruits and seeds as a storage carbohydrate (in the polymeric form), accounting for its presence in higher concentrations than the other sugars quantified. Both glucose and cellobiose in the extracts are possible byproducts of cellulose breakdown during extraction [44]. The fruit pulp and seeds and their extracts may be a valuable source of saccharides. This is in contrast to refined sugars, whose consumption results in a rapid and significant increase in blood glucose. Sugars found in most whole fruits cause a gradual increase in blood glucose as the fruit is mechanically digested in the presence of soluble fibres. This causes blood sugar levels to rise slowly and steadily, resulting in a controlled insulin response [45]. Furthermore, whole fruits contain essential nutrients such as vitamins, minerals, and phytonutrients, while refined sugars do not.

Organic acids are important compounds in fruits that contribute to their taste and aroma. Organic acids primarily contribute to the sour taste and overall flavour profile of fruits, which can influence the sensory perception alongside aromatic compounds. While organic acids themselves generally have limited direct impact on aroma, they often act synergistically with volatile aromatic compounds, such as those identified in Table 3, to shape the fruit’s overall aroma profile [46].

The HPLC analysis revealed significant levels of citric, lactic, and acetic acids in the extracts. Citric acid, commonly used in food products as a flavouring and preservative agent [47], has been reported in other studies to exhibit antioxidant properties and potential benefits related to inflammation and oxidative stress in animal models [48]. Lactic acid is known for its role in food preservation and fermentation processes, and some studies suggest it may support intestinal health [49]. Acetic acid, a component of vinegar, has also been associated with effects on digestion and metabolism in experimental studies [50]. However, it is important to note that the presence of these acids in our samples does not directly indicate health benefits in humans, and further research is needed to evaluate their bioavailability and physiological impact.

Future research should focus on evaluating the bioavailability and metabolism of these compounds following ingestion. Dose–response studies and pharmacokinetic analyses would help determine effective concentrations and biological fate. Ultimately, well-designed clinical trials are essential to confirm any health-promoting properties in humans and to assess safety and efficacy under relevant consumption conditions.

More detailed GC–MS analysis of methanolic, terpene-rich extracts revealed the high content of lanosterol, cholesterol, and stigmasterol derivatives in *S. cinereum*. The applications and utilization of these phytochemicals in a variety of fields are gaining popularity, particularly in the food industry, because their consumption has been linked to human health benefits such as anti-aging, anti-cancer, antioxidant, and anti-atherogenic, among others [51]. Equivalent lanosta-8,24-dien-3-ols include euphol, tirucallol, and lanosterol, as well as their isomers and derivatives, particularly acetates, solvates, and hydrates. These compounds had previously been isolated from Euphorbiaceae plants. Lanosta-8,24-dien-3-ols have been shown to inhibit enzymes associated with type-C protein kinases, which reduces cancer cell proliferation. In addition to tumour treatment, lanosterols are effective against inflammation and pain, with no known side effects. Another triterpenoid identified in methanolic extracts was α-amyrin acetate. This compound was also detected in wild *Scolymus maculatus* [52]. Its high free radical scavenging and resulting antioxidative properties were confirmed by the DPPH assay. It also demonstrated antibacterial activity in previous studies.

Some interesting compounds, such as friedelin and chromane, were also found, albeit in smaller amounts. Friedelin and its derivatives, also known as friedelane triterpenoids, have antimicrobial, anti-cancer, and anti-HIV properties [53]. Chromane is a structural component in more complex compounds, such as E vitamins. The chromanol ring system has also received much attention recently as a valuable pharmacophoric scaffold, especially since the discovery of tocochromanols, lipid-soluble compounds known as vitamin E [54]. Vitamin E is a free radical scavenger with significant potency against oxidative stress, which is linked to Alzheimer’s disease [55]. In *S. cinereum*, a diterpenoid geranylgeranyl alcohol was found in the fruit pulp. This is produced through the isoprenoid pathway and can be phosphorylated to yield geranylgeranyl pyrophosphate (GGPP), a precursor to geranylgeranylation, a type of protein prenylation. GGPP also serves as an intermediate in the synthesis of vitamins A, E, and K [56]. The presence of geranylgeranyl alcohol in the extract indicates the presence of these vitamins in the fruits, which are potent antioxidants with numerous other advantages [57]. Another antioxidative phytocomponent—naphthoquinone acetyl codeine, detected in *S. cinereum* seed extract—is a compound that is gaining more attention due to its anti-cancer activity [58]. Similarly, the identified ethyl ester of 2-acetylamino-5-iodo-4-p-tolyl-thiophene-3-carboxylic acid has been proven in other studies to have anti-cancer and antimicrobial properties [58].

The antioxidant activities of *S. cinereum* extracts were evaluated using DPPH, ABTS, and FRAP assays, with results indicating significant radical scavenging and reducing capacities. Notably, the acetone extract of pulp exhibited the highest DPPH activity (4.29 mg TE/g DM), while the water extract of seed demonstrated the highest FRAP value (6.20 μM Fe^2+^/g DM). These findings are consistent with those reported for *Sideroxylon lanuginosum*, which also exhibited notable antioxidant activities [59].

The antioxidant and oxidoreductive activity of a plant are largely influenced by the amount of phenolic compounds it contains. The results of the antioxidant and oxidoreductive activity of *S cinereum* fruit and seed extracts are similar to reported findings [60]. The antioxidant effects of different solvent extracts were concentration dependent, reflecting the solubilization properties of antioxidative compounds. Thus, the seeds were rich in hydrophilic substances, which have a high antioxidant capacity, while fruits’ antioxidative potential was exerted mainly from hydrophobic compounds. Our results are consistent with the studies conducted on *Dipsacus asperoides* [61] and *Olea europaea* [29] that show similar relations.

Our observations are confirmed by studies on Artocarpus odoratissimus (called Terap), where ethanol extracts from the seeds of this plant contained significant amounts of hydrophilic polar compounds, such as phenolic acids (such as chlorogenic acid), 4-pyrone derivatives (such as kojic acid) and salicylic acid derivatives, such as aminosalicylic acid (4-Aminosalicylic acid, para-aminosalicylic acid) and 5-hydroxymethylfurfural (5-HMF) [62].

A number of studies indicate the presence of hydrophobic (lipophilic) antioxidants in the fruits of tropical plants, such as carotenoids, which include β-carotene, β-cryptoxanthin, lycopene and lutein.

The presence of these substances in significant amounts was confirmed in the pulp of fruits such as green star apple (*Caimito verde*) and purple star apple (*Caimito morado*) belonging to the species *Chrysophyllum cainito* L.—a tree originating from the Caribbean region, and in the pulp of fruits such as yellow cashew (*Maranon amarillo*) and red cashew (*Maranon rojo*) belonging to the species *Annacardium occidental*—an evergreen tree from tropical areas of South America [63].

Significantly low and even negative FRAP correlations were observed between the studied TPC, TFC and antioxidant results, indicating that these compounds were not the main contributors to the antioxidant activities of the extracts. In fact, the TTC R values were the highest, especially with ABTS. This indicates that the terpenes of *S. cinereum* have a greater antioxidant capacity than its phenolic compounds due to a higher yield. Terpinc et al. [64] studied the correlation between antioxidant properties and the TPCs and also observed a statistically significant negative correlation between TPC and reducing power, with a free radical scavenging activity R > 0.75. Similarly, significant negative correlations were observed between TPC and reducing potential FRAP values in studies on the leaves and roots of sorrel (*Oxalis corymbosa*) [65]. Various plant phytochemicals can react with the Folin–Ciocalteu reagent, including organic acids. Low TPC values might be due to the specific nature of the antioxidant compounds present in the fruits or extracts [66].

To summarise, the variation in antioxidant activity observed among *S. cinereum* extracts is likely influenced by differences in phytochemical composition, which in turn are affected by both the plant part and the extraction solvent.

Previous studies have shown that classes of phytochemicals such as phenols, flavonoids, and tannins are major contributors to antioxidant capacity due to their ability to donate electrons or hydrogen atoms to neutralize free radicals.

The elevated FRAP values in acetone pulp extracts and high DPPH activity in propanol seed extracts may therefore be attributed to the higher concentrations or bioavailability of such antioxidant-rich compounds in those fractions.

The strong reducing capacity of the seed water extract may be linked to specific hydrophilic phenolic compounds known to be abundant in seeds, which often serve protective roles during seed dormancy and germination. This extract’s performance highlights the potential of aqueous extraction methods for isolating bioactive compounds from plant seeds, which could be advantageous for food applications where organic solvent use is restricted.

Overall, the correlation between solvent type, phytochemical solubility, and antioxidant performance underscores the importance of solvent selection in maximizing extraction efficiency of bioactive compounds. Future studies should include quantitative profiling that better delineate the specific contributions of individual phytochemicals to antioxidant effects.

The results suggest that *S. cinereum* fruits may be a potential natural source of terpenes, antioxidants, and other bioactive phytochemicals with numerous applications. For this reason, *S. cinereum* fruit and seed extracts can be treated as potential raw materials for the food industries. Because of the growing prevalence of diabetes in Mauritius and throughout the world, plant products such as low-calorie, flavourful teas with health advantages such as anti-diabetic and antioxidant qualities are gaining popularity in the plant product industry. The study gives positive data for the creation of such goods from endemic plants, while also promoting the Mauritian flora.

The spectrophotometric analyses we used provided general and preliminary information on selected groups of bioactive substances and antioxidant activity. An important confirmation of the results obtained by us from spectrophotometric methods is the GC–MS analysis (gas chromatography coupled with mass spectrometry), on the basis of which we confirmed the presence of, among other things, geranylgeranyl alcohol, in the pulp of *S. cinereum* fruit. This is a chemical substance belonging to the group of diterpenoids, which, due to the presence of a hydroxyl group and alternating single and double bonds (conjugated systems), shows strong antioxidant properties. The presence of geranylgeranyl alcohol may explain the high antioxidant activity in methanol solution measured by the ABTS method, where, among many antioxidants, this substance could effectively reduce the ABTS•+ cation radical to the unreactive form ABTS (2,2′-azino-bis(3-ethylbenzothiazoline-6-sulfonic acid) [67].

Another substance whose presence in significant amounts was confirmed by GC–MS analysis in the pulp of *S. cinereum* fruit is α-amyrin, containing a double bond between positions 12 and 13, in which the hydrogen in position 3 beta is substituted with a hydroxyl group [68].

Research results indicate that this substance, belonging to pentacyclic triterpenoids, has strong antioxidant properties, thanks to which it can effectively neutralize ABTS cation radicals (ABTS•+) [69].

As a result of the GC–MS analyses, the presence of morphinan-6-ol in *S. cinereum* seeds was confirmed. Studies confirm that this compound, belonging to the morphinates (a group of alkaloids with a four-ring structure), contains a hydroxyl group (–OH) at the carbon atom in position 6 of its structure, which means that morphinan-6-ol may exhibit significant antioxidant properties [70].

The ability of morphinan-6-ol to scavenge free radicals may also be due to the phenolic structure of this compound, which is largely similar (based on the presence of aromatic rings with a hydroxyl group (–OH) to the structure of the basic carbon skeleton of polyphenolic compounds [71,72].

## 4. Materials and Methods

### 4.1. Fruit Sampling

**Figure 6:**
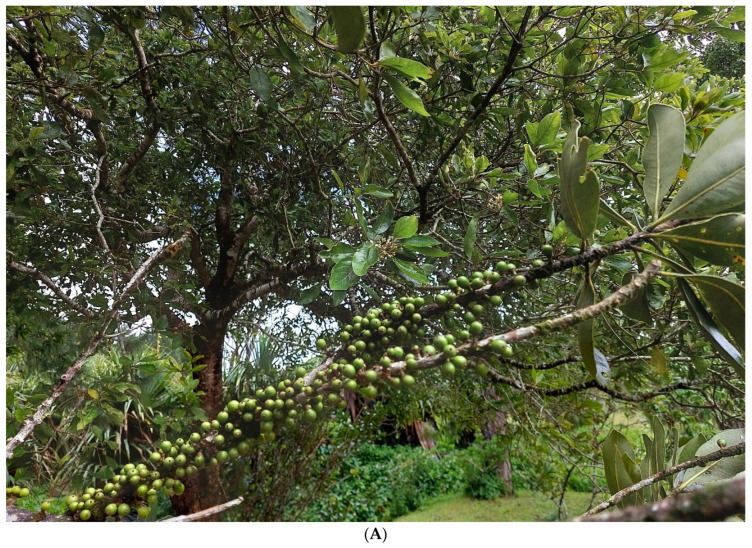

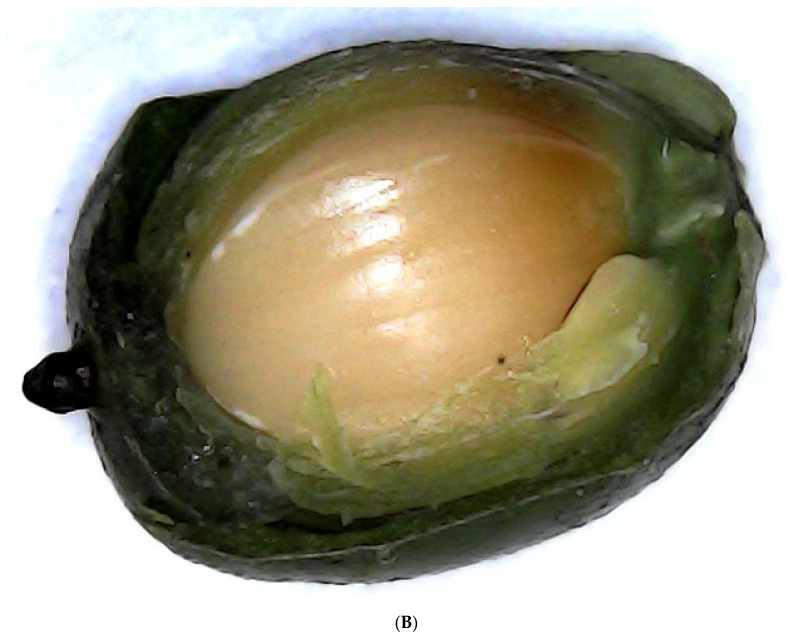
*Sideroxylon cinereum* on branch (**A**) and a cross section of its fruit (**B**).

The plant fruit (Figure 6) collection took place at the Native Plant Propagation Centre, which is located in the central region of Mauritius (Curepipe, Plaines Wilhems) and benefits from a mild tropical maritime climate all year (latitude 20.3484, longitude 57.5522, altitude 151 m). Initial visits were made to identify plants and to present the research’s intentions and goals. Prior to visits, permission was obtained from Mauritius’ Ministry of Agro-Industry, Food Production, and Security’s National Parks and Conservation Service. The sampling was conducted in October, a summer month in Mauritius.

### 4.2. Reagents and Chemicals

The lead acetate, ammonia, chloroform, sulphuric acid, iron (III) chloride, sodium hydroxide, iodine and potassium iodide (Wagner’s reagent), copper (II) sulphate, sodium citrate and anhydrous sodium carbonate (Benedict’s reagent) for the general qualitative analysis of bioactive groups present in the extracts were supplied by Acculab Ltd. (Nouvelle France, Grand Port, Mauritius). The following standards and reagents were used for the spectrophotometric methods: gallic acid, linalool, quercetin, iron(II) sulphate, Trolox (6-hydroxyl-2,5,7,8-tetramethylchromo-2-carboxylic acid), Folin–Ciocalteu reagent, calcium carbonate, sodium nitrate, aluminium chloride, DPPH (2,2-diphenyl-1-picrylhydrazyl), ABTS (2,2-azino-bis(3-ethyl benzothiazoline-6-sulphonic acid)) and TPTZ (2,3,5-triphenyltetrazolium chloride) and methanol, ethanol, propanol and acetone (Pol-Aura, Zabrze, Poland). Standards for HPLC included glucose, sucrose, fructose, rhamnose, xylose, galactose, mannose, arabinose, oxalic acid, citric acid, isocitric acid, malic acid, tartaric acid, malonic acid and succinic acids (Sigma Aldrich—Merck, St. Louis, MO, USA). Furthermore, solvents such as acetonitrile, sulphuric acid (Pol-Aura, Zabrze, Poland), and HPLC-grade water were used as the mobile phase in this study to separate and quantify the targeted compounds.

### 4.3. Phytochemical Extraction

One kilogram of healthy mature fruits was chosen and harvested from a single tree. The seeds were separated from the pulp, both of which were finely cut into pieces and dried at a temperature of 50 °C for 48 h in a drier (Vindon Scientific, Rochdale, UK). The dried fruit was ground and sieved to produce a fine powder (<500 µm). A moisture analysis balance was used to determine the moisture content of the powder (MA-30, Sartorius Lab Instruments GmbH & Co. KG, Göttingen, Germany). Water, ethanol, methanol, propanol, and acetone were used as solvents to extract metabolites from the fruits. One gram of dried fruit powder was placed in a 10 mL test tube, followed by 10 mL of solvent (water, methanol, ethanol, propanol, acetone). The tubes were sealed, and a rotary shaker was used for agitation (06-MX-RD-PRO, Chemland, Stargard, Poland) at 70 rpm at room temperature. After 24 h, the tubes were centrifuged at 5000 rpm for 15 min at 15 °C (MPW-260, MPW MED. INSTRUMENTS, Warsaw, Poland). The supernatant was collected, the volume and weight were measured, and the samples were kept at 4 °C. All solvent extracts were used for qualitative phytochemical analysis, TPC, TFC and TTC. Water extract was used for reducing sugars and protein quantification. Water extract was equally used for HPLC analysis while methanol extract was used for GC–MS analysis.

### 4.4. Qualitative Phytochemical Screening

The general qualitative phytochemical analysis was carried out on all five solvent extracts according to Parbuntari et al. [73] and as shown in Table 6. This shows the phytochemicals tested, their test reagents and method, and the observation of a positive result.

### 4.5. Quantitative Phytochemical Analysis

#### 4.5.1. Total Phenolic Content (TPC) Determination

The stock gallic acid solution was prepared by dissolving 0.01 g of gallic acid in 10 mL of distilled water. Standard solutions of gallic acid were prepared by serial dilutions using distilled water (0-1000 μg/mL). To 20 μL of gallic acid solutions and extracts in a test tube, 1580 μL of distilled water was added, followed by 100 μL of Folin–Ciocalteu reagent, and the mixture vortexed. The mixture was incubated for 6 min at room temperature (25 °C). An amount of 300 μL of saturated NaCO_3_ solution was then added to the tube and vortexed thoroughly until a permanent blue colour was obtained. The resulting solution was incubated at 38 °C for 30 min in the dark, and the absorbance was measured at 765 nm compared with a blank (SEMKO, Warsaw, Poland). TPC was expressed as mg of gallic acid equivalent (GAE) per gram of plant material dry matter (DM) [74,75].

#### 4.5.2. Total Flavonoid Content (TFC) Determination

An amount of 5 mg of quercetin was dissolved in 1 mL of methanol to make a stock quercetin solution. Serial dilutions of quercetin (0-200 g/mL) were used to prepare standard solutions. An amount of 4 mL of distilled water was added to 1 mL of diluted standard quercetin solutions and 1 mL extracts. Furthermore, 0.3 mL of 5% NaNO_3_ solution was added. Following 5 min, 0.3 mL 10% AlCl_3_ solution was added. After 6 min, 1 mL of 1 M NaOH was added, and the total volume was brought up to 10 mL using distilled water and carefully mixed. After 20 min, the mixture was vortexed. The absorbance of the reaction mixtures was measured at 510 nm compared with a blank. TFC was expressed as mg of quercetin equivalent (QE) per gram of plant material DM [76].

#### 4.5.3. Total Terpenoid Content (TTC) Determination

A standard linalool solution with concentrations ranging from 0 to 200 µg/mL was prepared. An amount of 2 mL of chloroform was added to 1 mL of standard solutions and 1 mL of extracts. The sample mixture was thoroughly vortexed and left for 3 min. An amount of 200 μL of concentrated sulphuric acid was poured into the mixture and incubated in the dark for 2 h at room temperature. During incubation, a reddish-brown precipitate formed in the mixture. The supernatant was carefully decanted so that the precipitation was not disturbed. Furthermore, 3 mL of absolute methanol was added, and the mixture was vortexed thoroughly to dissolve the precipitate completely. At a wavelength of 538 nm, the reaction mixtures’ absorbance was measured compared with a blank. The terpenoid concentration was expressed as mg of linalool equivalent (LE)/g per gram of plant material DM [77].

#### 4.5.4. Reducing Sugar (RS) and Protein Content Determination

A 2 mg/mL stock glucose solution was made by dissolving 0.4 g of glucose in 200 mL of distilled water. Glucose standard solutions were prepared by serial dilutions in distilled water (0-1000 µg/mL). An amount of 4 mL of distilled water and 250 μL of 1% 3,5-dinitrosalicylic acid (DNS) reagent in 0.4 M NaOH were added to 0.5 mL of diluted standard glucose solutions and 0.5 mL of water extracts. The samples were thoroughly vortexed for proper mixing before being incubated in a boiling water bath for 5 min. The sample was cooled to 50–60 °C before being mixed with 3 mL of distilled water. The sample was vortexed once more, and the absorbance measured at 530 nm compared with a blank using a spectrophotometer (SEMKO, Warsaw, Poland). The reducing sugar concentration was expressed as mg of glucose equivalent (Glu) per gram of plant material DM [74,75].

For protein content determination, a stock solution of commercial bovine serum albumin (BSA) at a concentration of 2 g/L was prepared (2000 µg/mL). Plant material samples were powdered with liquid nitrogen using precooled mortars and pestles. Proteins were extracted by homogenization in a cold 0.05 M Tris buffer at a ratio of 100 to 300 mg fresh mass: volume buffer. An amount of 0.05 g of antioxidant polyvinyl polyproline was added to each sample during the homogenization procedure. Homogenates were transferred to cold 2 mL Eppendorf tubes and centrifuged at 4 °C at 14 000 rpm for 20 min. Sample tubes were kept on ice and closed until centrifugation to prevent oxidation. An amount of 0.1 mL of each sample supernatant and working standard solution were transferred to assay tubes. A blank extraction buffer of 0.1 mL was also prepared. The Bradford reagent (containing Coomassie blue dye) was then added in 3 mL increments to each tube. To avoid foam formation in the sample, the tubes were gently vortexed. After 5 min and before 1 h, the absorbance at 595 nm (vs. the blank) was measured spectrophotometrically, taking into account the protein–dye complex’s stability, and expressed as mg of protein per g of plant material DM [78].

### 4.6. Ultraviolet–Visible (UV–Vis) Spectroscopy

The extracts (3 mL) were placed in a quartz cuvette and scanned using an Ultrospec 2000 UV–Vis (Pharmacia Biotech, Uppsala, Sweden) between the wavelengths of 200 and 700 nm. The maximum values were recorded. This approach detects phytochemicals by recognising molecules with π-bonds, lone pairs of electrons, σ-bonds, aromatic rings, and chromophores in the UV–Vis portion of the electromagnetic spectrum [79].

### 4.7. Mid-Infrared (MIR) Spectroscopy

Finely ground freeze-dried plant powder was used for the FT–IR analysis. FT–IR/ATR spectra were recorded in the 4000–400 cm^−1^ range using a Nicolet 6700 spectrometer (Thermo Fisher Scientific, Waltham, MA, USA) with a portable ATR assembly. The resolution of these measurements was 2 cm^−1^. Each sample was measured in three replicates. The replicate spectra were compared and analysed using commercial computer software (OriginPro 2024b).

### 4.8. Gas Chromatography–Mass Spectrometry (GC–MS)

GC–MS was undertaken on the methanolic extracts. The procedure was carried out on Agilent GC–MSD (mass spectrometer 5973 with GC 6890 N, UK) fitted with a DB-5 column and 30.0 m × 250 µm × 1.00 µm capillary. An oven program started at 70 °C and ramped at a rate of 6 °C/min to 150 °C, held for 2 min, and then ramped to 290 °C and held for 12 min. The total run time was 50.67 min. The mass spectral scan was an EI scanning from 40.0 amu to 580.0 amu. The MS source temperature was 230 °C, MS quad was set at 150 °C inlet, and auxiliary temperatures were held at 250 °C and 280 °C, respectively. The bioactive constituents in the extracts were identified using commercial libraries (NIST 8th edition, Wiley and Cayman) and a comparison of mass spectra, matches percentage (>80%), and reference compound retention times.

### 4.9. Sugar and Organic Acid Determination by HPLC

HPLC was undertaken on the water extracts. The analysis was carried out using an HPLC system equipped with a pump (Knauer Smartline 1000,KNAUER Wissenschaftliche Geräte GmbH, Berlin, Germany), vacuum degasser (Knauer Smartline Manager 5000,KNAUER Wissenschaftliche Geräte GmbH, Berlin, Germany), autosampler (Knauer Azura AS 6.1 L, KNAUER Wissenschaftliche Geräte GmbH, Berlin, Germany) and detector (Knauer RI detector K-2300, KNAUER Wissenschaftliche Geräte GmbH, Berlin, Germany). Separation of saccharides was carried out using the column Phenomenex PhenoSphere^TM^ NH2 (Phenomenex, Torrance, CA, USA) 150 × 4.6 mm, eluent: MeCN (acetonitrile): water (85:15 *v*/*v*), temperature: 40 °C, flow rate: 1.0 mL/min. Separation of organic acids was carried out using the column Phenomenex Rezex^TM^ ROA Organic Acid H+ (Phenomenex, Torrance, CA, USA) 300 × 7.8 mm, eluent: 5 mM H_2_SO_4_, temperature: 60 °C, flow rate: 0.6 mL/min. Software Clarity Chrome was the data handling system. Saccharides and organic acids in samples were identified by comparing the resulting chromatograms to those obtained from a standard mixture. Retention time matching was used to identify the peaks. To quantify each sugar and organic acid, the peak area obtained for a specific compound in the examined samples was compared with that of standard solutions.

### 4.10. Antioxidant and Oxidoreductive Activity Determination

#### 4.10.1. Ferric Reducing Antioxidant Power (FRAP) Assay

By dissolving 0.0015 g of FeSO_4_ in 10 mL of distilled water, a stock FeSO_4_ solution with a concentration of 1 mM was prepared. Serial dilutions of the FeSO_4_ (0–200 mol/dm^3^) were used to prepare standard solutions. A tube was filled with 1000 μL of FRAP working solution. An amount of 35 μL of the test sample was added, and the mixture was thoroughly mixed and incubated at 36 °C for 15 min. The absorbance of the reaction mixtures was measured at a wavelength of 593 nm compared with a blank. The test samples’ reducing activity was expressed as mol of iron (II) sulphate (FeSO_4_)/g per gram of plant material DM [74,75].

#### 4.10.2. 2,2′-Azino-bis(3-ethylbenzothiazoline-6-sulfonic Acid) (ABTS) Assay

Stock Trolox solution was prepared by dissolving 0.05 g Trolox in 5 mL distilled water. Trolox standard solutions were prepared through serial dilutions in distilled water (100–600 μM). In a cuvette, 1000 μL of ABTS working solution and 20.4 μL of the test sample were added, the stopwatch was activated, and the mixture was thoroughly mixed for 10 s. A spectrophotometer was used to measure the absorbance of the reaction mixtures against a blank at a wavelength of 734 nm. The capacity of the test samples to scavenge free radical cations was expressed as mg Trolox equivalent (TE)/g per gram of plant material DM [74,75].

#### 4.10.3. 2,2-Diphenyl-1-Picrylhydrazyl (DPPH) Assay

Stock Trolox solution was prepared by dissolving 0.06 g of Trolox in 250 mL of dis-tilled water. Trolox standard solutions were prepared through serial dilutions in distilled water (0–400 mg/mL). A tube was also filled with 1000 μL of DPPH working solution. An amount of 34.5 μL of the test sample was added, thoroughly mixed, and incubated in the dark for 20 min at room temperature. The absorbance of the reaction mixtures was measured at 517 nm against a blank. The test samples’ reducing activity was expressed as mg Trolox equivalent (TE)/g per gram of plant material DM [74,75].

### 4.11. Statistical Analysis and Correlation

The experimental data were processed and analysed in Microsoft Excel 2019, and the results were expressed as the mean, standard deviation of three analytical triplicates. Standard curves were plotted using linear regression to obtain a line of best fit. One-way ANOVA, undertaken with Statgraphics Centurion 19 (Statgraphics Technologies, Inc., The Plains, VA, USA) statistical software, yielded significant differences between variables. Statistical significance was defined as a *p*-value of 0.05. Pearson correlation analysis established a link between polyphenol, flavonoid, and terpene content and antioxidant activity. Statistical analysis was performed using one-way ANOVA followed by post-hoc testing to determine significant differences between compounds within each extract type, with statistical significance defined as *p* < 0.05.

## 5. Conclusions

The Mauritian endemic fruit *S. cinereum* and its seeds, according to the findings of the current study, are a rich source of basic compounds and phytochemicals with phyto nutritional potential. The qualitative and quantitative analyses confirmed the content of a significant amount of proteins and sugars, which are important biocomponents due to their nutritional value. The organic acids detected in the obtained extracts are known to possess several health benefits, including lower blood cholesterol levels. Both plant parts, fruits, and seeds were found to be rich in terpenes, saponins, coumarins, tannins, sulphoxide, and morphinan alkaloids.

Among terpenes, predominantly lanosterol, stigmasterol, and cholesterol derivatives were detected. These diverse groups of phytochemicals show many health-promoting activities, including anti-cancer, antibacterial, and antioxidative. The antioxidative and oxidoreductive tests correlated with terpenes content confirmed that this group of compounds is involved in antioxidative activity.

The phenolic compound content in plant-based foods plays an equally critical role in determining their potential health benefits, largely due to the well-established correlation between phenolic levels and antioxidant capacity. Given this association, plant extracts rich in phenolic compounds are increasingly being considered for therapeutic applications in oxidative stress-related conditions, such as diabetes and cancer. Accurate quantification and characterization of these compounds are therefore essential to fully harness the medicinal potential of plant materials.

While this study has contributed to the understanding of the major phytochemicals present in fruit extracts, several areas warrant further investigation. Notably, the methanolic pulp extract exhibited a particularly high phenolic content, and elevated terpene concentrations were observed across most samples. These findings highlight the need for more detailed profiling of individual phenolic constituents. This would allow for even more detailed results by eliminating the potential risk of overestimating the results of total polyphenolic compounds, which may be caused by the interaction of, among others, organic acids with the Folin–Ciocalteu reagent.

Future research should focus on the isolation and structural elucidation of these compounds to better understand their specific contributions to the overall antioxidant activity. Such efforts could provide deeper insights into the bioactive properties of these extracts and support their potential development into functional agents.

## Figures and Tables

**Table 1:** Qualitative phytochemical composition of extracts.

Phytochemicals	Solvents									
	Water		Methanol		Ethanol		Propanol		Acetone	
	Pulp	Seeds	Pulp	Seeds	Pulp	Seeds	Pulp	Seeds	Pulp	Seeds
Phenols	+	−	++	+	++	+	+	+	+	+
Flavonoids	−	−	−	+	−	+	−	+	−	+
Terpenes	−	−	++	−	++	−	++	−	++	−
Steroids	−	+	++	++	++	++	++	++	++	++
Saponins	+	+	++	++	++	++	+	+	+	+
Coumarins	−	−	+	++	+	++	+	+	+	+
Alkaloids	+	−	++	++	++	++	+	+	+	+
Tannins	−	−	+	++	+	++	+	+	+	+
Reducing sugars	+	+	−	−	−	−	−	−	−	−
Proteins	+	+	−	−	+	+	−	−	+	+

++: Highly present, +: Low, −: Absent.

**Table 2:** Calculated concentrations of organic acids and saccharides in extracts and dried fruit.

Retention Time [min]	Acids Saccharides	Content	
		In Extract, mg/mL	In Fruit, mg/g (Dry Mass)
*S. cinereum*—pulp			
8.47	Citric	1.56 ± 0.08 ^F,b^	15.63 ± 0.78 ^E,b^
9.08	Tartaric	0.26 ± 0.01 ^BC^	2.65 ± 0.13 ^B^
10.15	Malic	0.18 ± 0.01 ^B^	1.84 ± 0.09 ^B^
12.16	Succinic	0.23 ± 0.01 ^B^	2.33 ± 0.11 ^B^
13.26	Lactic	1.41 ± 0.07 ^F,b^	14.12 ± 0.71 ^E,b^
15.55	Acetic Xylose	0.36 ± 0.02 ^D,b^ 0.03 ± 0.00 ^A^	3.64 ± 0.18 ^C,b^ 0.35 ± 0.02 ^A^
4.59			
5.56	Fructose	0.54 ± 0.03 ^E,b^	5.35 ± 0.27 ^D,b^
6.79	Glucose	3.91 ± 0.20 ^G,b^	39.09 ± 1.95 ^F,b^
*S. cinereum*—seeds			
8.54	Citric	0.73 ± 0.04 ^D,a^	7.29 ± 0.36 ^D,a^
13.34	Lactic	0.57 ± 0.03 ^C,a^	5.68 ± 0.26 ^C,a^
15.69	Acetic	0.25 ± 0.01 ^B,a^	2.46 ± 0.12 ^B,a^
5.50	Fructose	0.11 ± 0.01 ^A,a^	1.10 ± 0.01 ^A,a^
7.02	Glucose	1.16 ± 0.01 ^E,a^	11.60 ± 0.58 ^E,a^

Lowercase letters indicate statistically significant differences between pulp and seed for the same compound (*p* < 0.05). Uppercase letters indicate statistically significant differences between compounds within the same extract type (*p* < 0.05).

**Table 3:** Composition of phytochemical compounds in *S. cinereum* flesh and *S. cinereum* seeds.

Peak Number	Retention Time (min)	Peak Area (%)	Corresponding Compound	Common Name
*S. cinereum* pulp				
1	37.554	0.64	3,7,11,15-tetramethyl-2E,6E,10E,14-hexadecatetraen-1-ol	Geranyl alcohol
2	38.918	1.28	(3β)-cholest-5-en-3-ol	Cholesterol
3	41.319	0.44	Stigmasta-7,25-dien-3-ol, (3.beta,5.alpha.)-	delta-7,25-stigmastadienol
4	41.350	0.57	5-Chloro-2-iodobenzoic acid	5-Chloro-2-iodobenzoic acid
5	41.964	1.84	Ethanone, 1,1′-(6-hydroxy-2,5-benzofurandiyl)bis-	Euparone
6	42.149	0.47	Lanosta-9(11)-en-12-one	Parkeol
7	42.658	1.32	9,19-Cyclolanost-24-en-3-ol, (3. beta.)-	Cycloartenol acetate
8	42.818	1.14	Trimethylsilyl ether	Nalmefene
9	43.463	8.14	12-Oleanen-3-yl acetate, (3.α)-	α-amyrin
10	43.653	10.97	Lanosta-9(11),24-dien-3-ol, acetate, (3.beta.)-	Perkeol acetate
11	43.666	8.83	Octamethylicosahydropicen-3(2H)-one	Friedeline
12	44.440	52.58	Lanosta-8,24-dien-3-ol, acetate, (3.beta.)-	Lanosterol acetate
13	48.241	3.07	Dammara-20,24-dien-3-ol, 3-acetate, (3.beta)	Dammaradienyl acetate
14	48.911	8.71	2,2,5,7,8-Pentamethyl-chromane-6-sulfonyl chloride	Chromane
*S. cinereum* seeds				
1	34.772	22.41	1,2-Benzenedicarboxylic acid	Phthalic acid
2	35.810	0.50	4,5-epoxy-3-methoxy-17-methyl-	Morphinan-6-ol
3	38.899	7.31	26-Nor-5-cholesten-3.beta.-ol-25-one	Norcholesterol
4	41.350	2.00	33-Norgorgosta-5,24(28)-dien-3-ol, (3.beta.)-	33-Norgorgosta-5,24(28)-dien-3-ol, (3.beta.)-
5	41.369	1.01	2-Acetylamino-5-iodo-4-p-tolyl-thiophene-3-carboxylic acid ethyl ester	
6	41.921	0.54	4-Nitrophenyl-N-(2-chloroethyl)-N-nitrosocarbamate	Carbamic acid
7	42.615	0.48	Acetyl Codeine	Acetyl codeine
8	43.340	0.49	2,3,5,6-Tetrachloroiodobenzene	
9	43.463	7.34	4-Allyl-5-pyridin-3-yl-2,4-dihydro-[1,2,4]triazole-3-thione	
10	43.635	9.42	Lanosta-7,9(11)-dien-3-ol, acetate, (3.beta.)-	
11	44.378	45.67	Lanosta-8,24-dien-3-ol, acetate, (3.beta.)-	Lanosterol acetate
12	48.192	0.47	1,3-Benzenedicarboxylic acid, 5-nitro-, dimethyl ester	Dimethyl 5-nitroisophthalate;
13	48.825	0.53	2-[4-Acetamidophenylsulfonyl]-1,4 naphthoquinone	para-naphthoquinone
14	48.911	1.83	Benzo[1,2,5]oxadiazole, 5-(1H-benzoimidazol-2ylsulfanylmethyl)-	Benzo[1,2,5]oxadiazole, 5-(1H-benzoimidazol-2ylsulfanylmethyl)-

**Table 4:** The antioxidant properties of the extracts from the five different solvents of the pulp and seed of *S. cinereum*, along with their antioxidant values using the DPPH, ABTS and FRAP methods.

*S. cinereum*	Solvent	DPPH (mg TE/g DM)	ABTS (mg TE/g DM)	FRAP (uM Fe^2+^/g DM)
Pulp	Water	3.11 ± 0.07 ^b^	2.03 ± 0.26 ^a^	0.55 ± 0.39 ^a^
	Methanol	3.39 ± 0.21 ^b^	4.63 ± 0.28 ^b^	1.45 ± 0.39 ^b^
	Ethanol	3.17 ± 0.08 ^b^	4.52 ± 0.27 ^b^	2.57 ± 0.47 ^c^
	Propanol	4.29 ± 0.21 ^c^	5.41 ± 0.70 ^c^	3.27 ± 0.51 ^c^
	Acetone	2.40 ± 0.27 ^a^	4.44 ± 0.45 ^b^	4.69 ± 0.18 ^d^
Seed	Water	4.22 ± 0.53 ^bc^	2.71 ± 0.21 ^a^	6.20 ± 0.10 ^c^
	Methanol	3.95 ± 0.15 ^bc^	4.13 ± 0.31 ^b^	1.27 ± 0.24 ^a^
	Ethanol	3.77 ± 0.32 ^b^	4.36 ± 0.47 ^b^	1.63 ± 0.54 ^ab^
	Propanol	4.49 ± 0.22 ^c^	3.13 ± 0.05 ^a^	2.15 ± 0.02 ^b^
	Acetone	3.07 ± 0.07 ^a^	5.21 ± 0.18 ^c^	2.12 ± 0.28 ^b^

Lowercase letters mean statistically significant difference between solvent fractions (*p* = 0.05).

**Table 5:** Correlation between phytochemical contents and antioxidant properties of extracts.

	TPC	TFC	TTC	DPPH	ABTS	FRAP
TPC	1					
TFC	0.613863	1				
TTC	0.597528	0.87499	1			
DPPH	0.245845	0.152466	0.336171	1		
ABTS	0.081978	0.300145	0.480643	−0.15989	1	
FRAP	−0.36201	−0.3952	−0.36548	0.064946	−0.0111	1

**Table 6:** Qualitative tests for phytochemical screening.

Phytochemicals	Reagents and Method	Positive Result
Phenols	Few drops of Pb(C_2_H_3_O_2_)_2_ were added to a few drops of extract.	Appearance of a white precipitate.
Flavonoids	Few drops of 1% NH_3_ were added to a few drops of extract.	Appearance of a yellow coloration.
Steroids	Few drops of extract followed by few drops of CHCl_3_ and H_2_SO_4_ were added to the side of a test tube.	Formation of a reddish-brown ring at the interface.
Terpenes		Formation of yellow precipitate.
Saponins	Distilled water was added to a few drops of extract, and the mixture was shaken vigorously.	Formation of a froth, which remains for 15–30 min.
Alkaloids	To a few drops of extract, few drops of Wagner’s reagent were added.	Appearance of a reddish-brown precipitate.
Tannins	Few drops of 5% FeCl_3_ were added to a few drops of extract.	Appearance of a blue-black or brownish-green precipitate. Appearance of a blue-black or brownish-green precipitate.
Coumarins	To a few drops of extract, a few drops of NaOH were added.	Formation of a yellow colour.
Reducing Sugars	To 1 mL of extract, 2 mL of Benedict’s reagent was added and heated in a bath of boiling water for 3 to 5 min.	Development of a brick-red coloured precipitate.
Proteins	To a few drops of extract, 2 mL of NaOH and 5 to 6 drops of CuSO_4_ was added and the test tube gently shaken and allowed to stand for 4–5 min.	Appearance of a bluish-violet colour.

## Data Availability

No new data were created or analyzed in this study. Data sharing is not applicable to this article.
